# Micro-scale spatial metagenomics opens a new era in microbiome ecology

**DOI:** 10.1016/j.tim.2026.03.005

**Published:** 2026-08

**Authors:** Sarah Moraïs, Itzhak Mizrahi

**Affiliations:** 1 Department of Life Sciences, Ben-Gurion University of the Negev, Beer-Sheva 8410501, Israel; 2 The National Institute for Biotechnology in the Negev, Ben-Gurion University of the Negev, Beer-Sheva 8410501, Israel; 3 The Goldman Sonnenfeldt School of Sustainability and Climate Change, Ben-Gurion University of the Negev, Beer-Sheva 8410501, Israel

**Keywords:** spatial ecology, spatial structure, microbial interactions, microbial dynamics, microbial communities

## Abstract

Understanding microbial communities requires moving beyond 2D representations toward a holistic view that couples 3D spatial organization with ecological function, integrating microbial inventories, genes, expression profiles, and interactions at scales and dimensions in which microbial life unfolds. In this opinion article, we synthesize recent findings and emerging approaches that enable the investigation of microbial interactions within their native 3D context. We propose conceptual frameworks for integrating spatial–functional information into comprehensive ecological maps, providing new avenues to interpret microbial interactions and to test ecological theory *in situ*. Together, these insights outline a new ecological paradigm for microbiome research and highlight how spatially resolved understanding can be harnessed to interpret and ultimately guide the modulation of microbial interactions and ecosystem function in natural settings.

## Highlights

Spatial organization is a fundamental but long-overlooked dimension of microbial ecology that cannot be captured by conventional bulk metagenomic approaches.
Nano- to microscale cellular architectures constrain microbial interactions and functions, underscoring the need to extend spatial approaches from single cells to complex communities.
Emerging spatially resolved metagenomic methods, including metagenomic plot sampling by sequencing, Split-And-pool Metagenomic Plot-sampling sequencing, and micro-scale spatial metagenomics, now retain microbial spatial context while delivering sequencing-based taxonomic and functional resolution.
Together, both microscale cellular architectures and spatially resolved metagenomic technologies uncover micron-scale heterogeneity, spatial hubs, and strain-level organization within microbial communities that are invisible to spatially averaged data.
Spatially resolved ecological understanding provides empirical access to microbial biogeography at ecologically relevant scales, bridging theoretical and 2D understanding into real-world knowledge that could be harnessed to modulate microbial communities.

## Foundations of spatial microbial ecology

The foundations of spatial ecology can be traced back to Watt’s pioneering emphasis on spatial patterns in plant communities, which inspired the concept of the ‘shifting-mosaic steady state’ [1]. Subsequent decades saw critical advances in this area, whereby experimental studies highlighted the role of spatial refugia [2], theoretical work explored organismal diffusion through space in relation to community dynamics 3, 4, 5, and biogeographic frameworks in the 1950s and 1960s culminated in the theory of island biogeography 6, 7, 8 and the concept of metapopulations 9, 10. These milestones established space as a central dimension of ecological thought.

**Figure 1:**
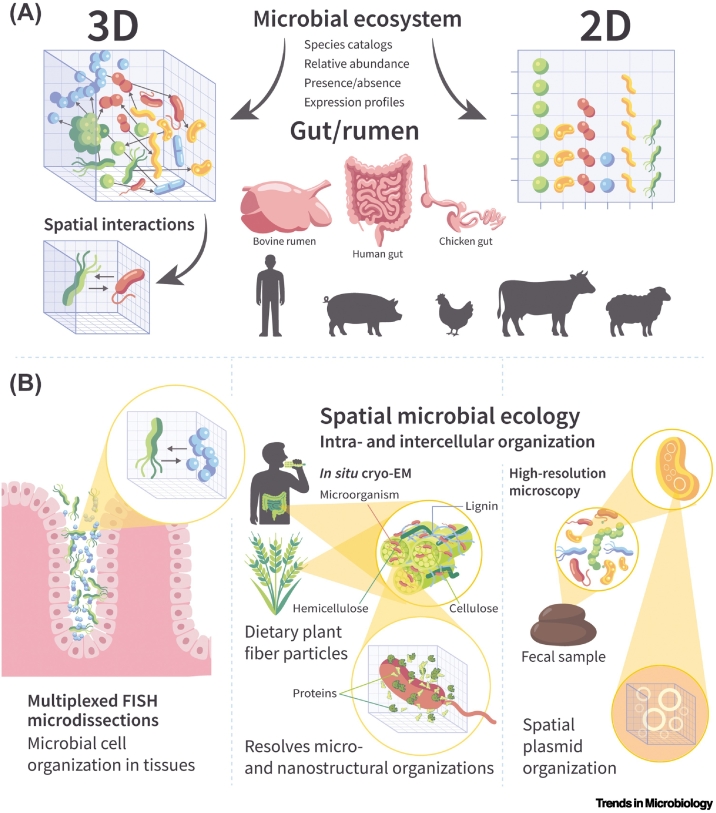
From conventional microbiome analysis to spatial microbial ecology. (A) Conventional microbiome approaches, including shotgun metagenomics, rely on averaged taxonomic and functional profiles that collapse 3D microbial ecosystems into 2D representations, whereas spatial microbial ecology preserves the spatial organization underlying microbial interactions. It is of note that although microbial communities are often structured in three dimensions, some systems, such as surface-associated biofilms, can exhibit predominantly 2D spatial arrangements. (B) Spatially resolved approaches, including imaging-based methods and cryo-electron tomography, reveal intra- and intercellular microbial spatial organization. EM: electron tomography; FISH: fluorescence *in situ hybridization.*

In microbial ecology, for more than a decade, shotgun metagenomics, metatranscriptomics, and metaproteomics have been instrumental in gut microbiome research, thus serving to illuminate the taxonomic and functional gene expression potential of microbial communities across hosts and environments [11]. These approaches have generated unprecedented catalogs of microbial genes, metabolic pathways, expression profiles, and strain-level diversity, revealing how microbiomes influence health, disease, and ecosystem functioning 12, 13, 14. Yet, a fundamental limitation has persisted: conventional approaches flatten and lump microbial ecosystems into bulk homogenized averages of species catalogs, genes, expression profiles, and other measurable aspects of microbial life—thereby erasing the spatial dimension that underpins ecological interactions and microbial organization (Figure 1).

Since microbes and their molecular machineries operate across nano- to micron scales, understanding their ecology requires approaches that capture spatial organization, as functions and interactions are inseparably tied to these microarchitectures. Techniques such as cryo-electron tomography (cryo-ET) now allow single-cell examination of microbial communities while revealing their micro- and nanostructural organization (Figure 1). Using this approach, recent studies have shown that bacterial populations exhibit distinct cellular architectures and phenotypic heterogeneity that support division-of-labor strategies during cellulose degradation [15]. Combined with proteomics, cryo-ET has provided insights into how keystone species fine-tune their specialized cell-bound enzymatic complexes for resistant starch breakdown in the human colon [16]. These studies underscore that microbial function is inherently governed by spatial constraints, reinforcing the need for approaches that capture microbial communities in their spatial ecological context. While cryo-ET has so far provided such insights mainly at the level of single microbes, the gap will be even greater when extending this understanding to the organization and interactions within complex communities. Integrating cryo-ET with emerging spatial proteomics approaches will offer a powerful avenue to link ultrastructural organization with protein localization and activity within microbial communities [17].

In any microbial ecosystem, the organization of species in space governs which microorganisms interact, how nutrients and other metabolites flow, and which lineages gain evolutionary advantages [18]. In the gut, nutrient gradients, host secretions, immune activity, and physical structures all carve the habitats into microniches [19]; therefore, the missing spatial dimension is fundamental, and collapsing microbial spatial organization into bulk averages obliterates the ecosystem’s natural ecology.

Early efforts to restore spatial context relied on either sampling distinct macrosites along the digestive tract—using techniques with varying spatial resolutions, such as biopsies or microdissection 20, 21, 22, 23, 24, 25, as exemplified by Zmora *et al*. in humans [26]. Complementary strategies have also used imaging-based approaches to directly visualize microbial organization *in situ* 27, 28 (Figure 1). Among these, fluorescence *in situ* hybridization (FISH)-based approaches, including multiplexed derivatives and signal-enhancement techniques, provide striking spatial views of microbial cells embedded within tissues and even of plasmids within individual microbial cells in natural settings (Figure 1). These methods reveal fine-scale colonization patterns, such as microbial organization along intestinal crypts, the layered structure of biofilms on surfaces, and even aspects of the intracellular organization of plasmids 27, 28, 29. These techniques offer micron-scale resolution and direct visualization of cell–cell proximities. More recently, multiplexed fluorescence methods have expanded the number of distinguishable taxa by layering dozens of probes into a single image 30, 31. Nevertheless, such techniques carry inherent limitations, with taxonomic coverage remaining partial and constrained by probe design and restricted functional inferences, leaving a substantial gap when compared to the breadth of taxa and diversity of functional pathways revealed by shotgun metagenomics. Consequently, the need for methods capable of delivering the taxonomic and functional breadth of sequencing while preserving spatial information at ecologically relevant scales has become essential.

## New generation of spatially resolved metagenomics

In 2019, Sheth *et al.* introduced Metagenomic Plot Sampling by Sequencing (MaPS-seq) [32], a groundbreaking attempt to bridge that gap. Their approach retained spatial arrangement by immobilizing intact gut microbiome samples in a gel matrix, cryofracturing them into micron-scale particles, and encapsulating these particles in droplets with barcoded sequencing. The result demonstrated a way to analyze which taxa co-occurred within the same microfragments of community structure, effectively preserving fine-scale spatial associations without relying on microscopy. MaPS-seq revealed that microbial distributions within mouse intestinal compartments are not random but spatially heterogeneous, featuring both coassociations and mutual exclusions across taxa. Moreover, it demonstrated that diet can reshape these fine-scale biogeographic patterns, altering which microbes share space and which are segregated. This sequencing method thus served to profile microbial biogeography at submillimeter resolution across hundreds of taxa, but relied on several technical steps that substantially limited its throughput, scalability, and accessibility.

Building on this conceptual advance, Richardson *et al.* recently introduced Split-And-pool Metagenomic Plot-sampling sequencing (SAMPL-seq) [33], a streamlined and scalable approach that applies the MaPS-seq principle to the human gut. By splitting and pooling micron-scale particles for barcoded sequencing, SAMPL-seq captures spatial colocalization at high throughput, enabling crossindividual comparisons at the microscale level. Applied to human fecal samples, SAMPL-seq revealed the existence of persistent spatial hubs—clusters of colocalized taxa dominated by families such as Bacteroidaceae, Ruminococcaceae, and Lachnospiraceae across individuals. Strikingly, dietary supplementation with inulin reorganized these hubs in a reversible manner, demonstrating that spatial microbiome structure can be diet-sensitive yet resilient. Where MaPS-seq established feasibility in mice, SAMPL-seq extended the framework into humans, offering statistical power and throughput that open the way for population-scale studies of spatial organization.

Most recently, Pietroni *et al*. have introduced micro-scale spatial metagenomics (MSSM) [34], a methodology that integrates cryosectioning, laser microdissection, and ultra-low-biomass sequencing to reconstruct microbial community structure and function at micron-scale resolution. Where MaPS-seq established that spatial heterogeneity could be captured by sequencing, and SAMPL-seq scaled that approach to high-throughput human studies, MSSM delivers functional and strain-level insight, prioritizing depth of information over throughput. Applied to the chicken gut, MSSM reveals striking fine-scale patterns: spatial segregation of taxa and functions within the colon, microdiverse strain distributions in the cecum, and patterns of SNP-level clustering invisible to bulk metagenomics. The picture that emerges is one of a richly textured microbial ecosystem, where even closely related strains partition space and where functional repertoires vary across micron-distanced microhabitats. Importantly, MSSM bridges the methodological gap between imaging-based approaches and bulk sequencing. MSSM is sensitive down to approximately 100 cells per sample, scales to hundreds of taxa and functional pathways, and preserves spatial context. Finally, the MSSM method is also modular, as cryosections can be multiplexed across other complementary spatially resolved techniques. These include recently developed spatial metabolomics approaches, such as matrix-assisted laser desorption ionization mass spectrometry imaging combined with *in situ* fluorescence labeling, which enable the visualization of metabolites and microbes at high spatial resolution and provide an additional layer of information on microbial interactions and metabolic exchange within complex ecosystems [35]. Integration with other spatially resolved modalities, including spatial transcriptomics 36, 37, spatial proteomics [17], and advanced microscopy (such as cryo-ET), would further enable an integrative ‘multi-omics cartography’ at scales directly relevant to microbial consortia and microniches. Together, MaPS-seq, SAMPL-seq, and MSSM exemplify a new generation of spatially resolved metagenomics—enabling both taxonomic and functional outputs.

## Harnessing spatial ecology modeling frameworks for 3D microbial data

The multifaceted relevance of space in species dynamics—in shaping resource utilization, movement, dispersal, and interactions among species—has long motivated the use of mathematical and statistical models. These models, ranging from process-based simulations to phenomenological regression approaches, provide essential tools for understanding how spatial structure drives ecological patterns. Their contributions extend to ecological forecasting and the estimation of the impact of environmental drivers on species distributions.

The growth of spatial modeling frameworks represents a quantum leap in ecological science. Early models considered space implicitly [38]; later ones captured it explicitly and realistically [39]. Frameworks range from discrete space units in cellular automata models to spatially explicit representations using *x*–*y* coordinates, which enable individual- or agent-based simulations of movement and connectivity [40]. By modeling processes across multiple spatial scales, spatially explicit models (SEMs) provide a comprehensive understanding of ecological dynamics, linking local interactions to landscape-level patterns, which are also applied to microbial communities [41].

Key concepts, such as spatial autocorrelation—the likelihood that nearby samples are more similar than expected by chance—are crucial for interpreting ecological data. Positive spatial autocorrelation reflects shared influences shaping organismal distributions, and its decline with distance illuminates the scale at which processes operate. Handling such data effectively has been enabled by SEMs [42], which simulate fine-scale landscape details and spatially dependent biological processes. SEMs stand in contrast to spatially implicit models [43], and their proliferation has sparked productive debates around realism, tractability, and generality.

From a technical standpoint, SEMs encompass diverse mathematical structures, including reaction–diffusion partial differential equations, patch models, cellular automata, and individual-based neighborhood models. Their value becomes evident when addressing small-scale contingencies, spatial heterogeneity, and deterministic interaction dynamics that often elude more abstract, spatially implicit approaches.

This long tradition of spatial modeling provides a valuable conceptual backdrop for interpreting the new wave of sequencing-based methods. MaPS-seq, SAMPL-seq, and MSSM can be seen as experimental analogs to SEMs: they generate empirical data that reveal the very fine-scale heterogeneities and spatial autocorrelations that theory has long predicted but rarely measured in microbial systems. Spatially resolved sequencing has the potential to transform our understanding of microbial ecosystems, echoing how metapopulation and island biogeography models have reshaped our view of macroscopic communities. A need for computational and analytical approaches applied to these high-dimensional datasets is now emerging.

## Toward predictive and interventional spatial microbiology

**Figure 2:**
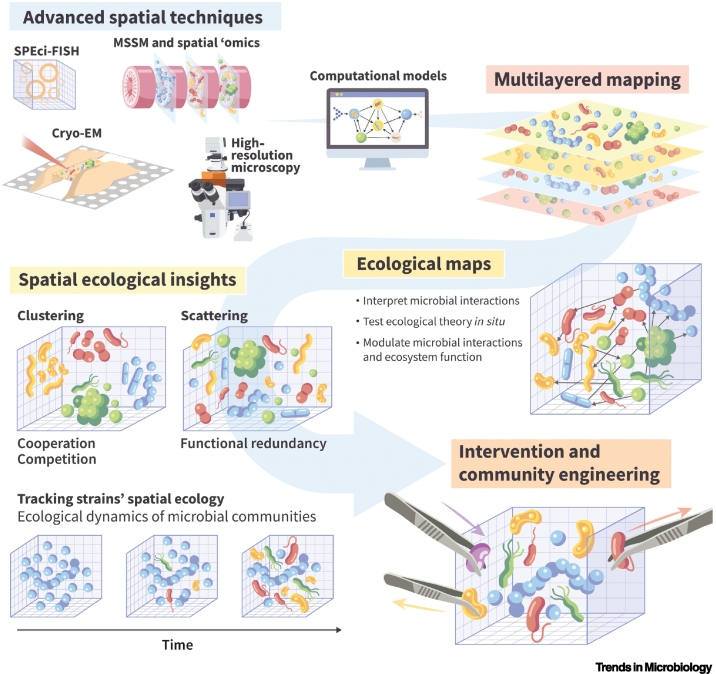
Toward a predictive and interventional framework for spatial microbial ecology. Spatially resolved metagenomics and complementary approaches (e.g., SPEci-FISH, cryo-EM, and high-resolution microscopy) coupled with computational models enable microbial ecosystems to be studied as structured, multilayered 3D landscapes. By mapping taxa, functions, and strain-level variation at micron scales, these methods enable the comprehension of microbial interactions and the testing of fundamental ecological principles, including spatial niche partitioning, cooperation and competition, functional redundancy, and ecological dynamics. Integrating spatial data with computational models provides a framework for ecological maps, ultimately enabling targeted interventions through manipulation of microbial spatial organization. EM: electron tomography; FISH: fluorescence *in situ hybridization; MSSM: micro-scale spatial metagenomics.*

Spatial data allow ecologists to test fundamental principles at microbial scales (Figure 2). How does spatial arrangement shape cooperation, competition, and evolution? How do communities partition niches within microns of space? Do closely related species exclude each other spatially to avoid competition, or do they cluster and cooperate? Do multiple taxa that carry the same pathway, scattered throughout a habitat, reduce spatial functional redundancy, or are they confined to specific microhabitats? Could tracking strain-level variants across microscale maps enable the study of evolutionary dynamics within microbial communities?

As with any technological advance, MSSM and other spatially resolved metagenomics, along with complementary methods such as spatial metabolomics [35] and spatial transcriptomics 36, 37, raise new challenges. Balancing microsample resolution with sequencing depth is a trade-off between more precise spatial maps with finer cuts but fewer reads per unit biomass. In addition, heterogeneous biomass distribution across tissues complicates normalization. Furthermore, integrating strain-resolved data into ecological frameworks requires computational models that can handle spatially explicit inputs (Figure 2). To address these limitations, cryosection-based MSSM could be combined with metabolite imaging, transcriptomics, and host gene expression profiling to create multilayered maps of host–microbe interaction zones (Figure 2). Comparative studies across hosts and environments could thus reveal whether spatial principles, such as clustering of syntrophic partners or segregation of competitors, are conserved across systems (Figure 2). Coupling spatial sequencing data to agent-based or reaction–diffusion models could yield predictive insights into emergent community properties by building on advances in computational ecology.

Ultimately, these methods invite a paradigm shift: microbial communities are not 2D but intricately textured. Recognizing and measuring texture, spatially and functionally, is essential to decipher the native ecology of microbes. The microbiome field is entering an era where microbial spatial locations are as important as their identity. Spatially resolved metagenomic methods provide complementary templates for studying microbial ecosystems as structured, dynamic landscapes rather than homogeneous ecosystems. Future steps should involve adapting these approaches across hosts and habitats, coupling them with metabolite and transcript distributions, and developing computational tools to model emergent properties from spatial data.

By making microbial spatial ecology experimentally tractable, spatially resolved metagenomics sets the stage for transformative insights into how microbial communities assemble, interact, and evolve at their native scales. Together, they shift microbiome research toward ecological maps that capture texture, structure, and dynamics, representing a significant conceptual advance (Figure 2).

## Concluding remarks and future perspectives

Importantly, these 3D ecological maps provide a powerful framework for moving from description to intervention in microbial ecology (Figure 2). By resolving how microbes are organized in space and how spatial proximity constrains interactions, resource exchange, and competition, these methods make it possible to identify spatial bottlenecks and interaction hubs that structure community function. Such insights enable the targeted modulation of microbial communities by reshaping spatial organization—for example, through controlled colonization, niche reconstruction, or manipulation of microenvironments—rather than by altering community composition alone. In this way, 3D perspectives open new avenues for steering microbial communities in a manner that aligns with their natural ecological dynamics.

Outstanding questions How does microbial community composition determine spatial organization at micron scales, and which taxonomic or functional traits most strongly shape spatial patterning?

What spatial principles govern microbial community assembly at micron scales, and are these principles conserved across hosts, body sites, and environments?

How does fine-scale spatial organization shape microbial cooperation, competition, and metabolic crossfeeding within complex communities?

Do closely related microbial strains spatially segregate to reduce competition, or colocalize to enable functional redundancy and resilience?

How stable are spatial microbial architectures over time, and how do perturbations such as diet, antibiotics, or inflammation reshape microscale biogeography?

To what extent does spatial heterogeneity influence functional output, including metabolite production, enzymatic activity, and host–microbe interactions?

Can spatially resolved metagenomics be integrated with metabolomics, transcriptomics, and imaging to generate unified, multilayered maps of microbial ecosystems that can be leveraged to modulate microbial communities?

Alt-text: Outstanding questions

## References

[1] Watt A.S. (1947). Pattern and process in the plant community. J. Ecol..

[2] Huffaker C.B. (1958). Experimental studies on predation: dispersion factors and predator-prey oscillations. Hilgardia.

[3] Hastings A., Gross L.J. (2012). Encyclopedia of Theoretical Ecology.

[4] Hilborn R. (1979). Some long term dynamics of predator-prey models with diffusion. Ecol. Model..

[5] Skellam J.G. (1951). Random dispersal in theoretical populations. Biometrika.

[6] Preston F.W. (1948). The commonness, and rarity, of species. Ecology.

[7] MacArthur R.H., Wilson E.O. (1963). An equilibrium theory of insular zoogeography. Evolution.

[8] MacArthur R.H., Wilson E.O. (1967).

[9] Hanski I. (1999). Metapopulation Ecology.

[10] Levins R. (1969). Some demographic and genetic consequences of environmental heterogeneity for biological control. Bull. Entomol. Soc. Am..

[11] Quince C. (2017). Shotgun metagenomics, from sampling to analysis. Nat. Biotechnol..

[12] Almeida A. (2019). A new genomic blueprint of the human gut microbiota. Nature.

[13] Stewart R.D. (2019). Compendium of 4,941 rumen metagenome-assembled genomes for rumen microbiome biology and enzyme discovery. Nat. Biotechnol..

[14] Sasson G. (2022). Metaproteome plasticity sheds light on the ecology of the rumen microbiome and its connection to host traits. ISME J..

[15] Tatli M. (2022). Nanoscale resolution of microbial fiber degradation in action. Elife.

[16] Wimmer B.H. (2025). Spatial constraints drive amylosome-mediated resistant starch degradation by *Ruminococcus bromii* in the human colon. Nat. Commun..

[17] Lundberg E., Borner G.H.H. (2019). Spatial proteomics: a powerful discovery tool for cell biology. Nat. Rev. Mol. Cell Biol..

[18] Tropini C. (2017). The gut microbiome: connecting spatial organization to function. Cell Host Microbe.

[19] Donaldson G.P. (2016). Gut biogeography of the bacterial microbiota. Nat. Rev. Microbiol..

[20] Eckburg P.B. (2005). Diversity of the human intestinal microbial flora. Science.

[21] Nava G.M. (2011). Spatial organization of intestinal microbiota in the mouse ascending colon. ISME J..

[22] Riva A. (2019). A fiber-deprived diet disturbs the fine-scale spatial architecture of the murine colon microbiome. Nat. Commun..

[23] Sbardellati D.L. (2020). The bovine epimural microbiota displays compositional and structural heterogeneity across different ruminal locations. J. Dairy Sci..

[24] Li M. (2009). Effects of sampling location and time, and host animal on assessment of bacterial diversity and fermentation parameters in the bovine rumen. J. Appl. Microbiol..

[25] Dubinsky V. (2023). Strains colonizing different intestinal sites within an individual are derived from a single founder population. mBio.

[26] Zmora N. (2018). Personalized gut mucosal colonization resistance to empiric probiotics is associated with unique host and microbiome features. Cell.

[27] Pédron T. (2012). A crypt-specific core microbiota resides in the mouse colon. mBio.

[28] Valm A.M. (2012). CLASI-FISH: principles of combinatorial labeling and spectral imaging. Syst. Appl. Microbiol..

[29] Zorea A. (2025). ProFiT-SPEci-FISH: a novel approach for linking plasmids to hosts in complex microbial communities at the single-cell level. Microbiome.

[30] Cao Z. (2023). Spatial profiling of microbial communities by sequential FISH with error-robust encoding. Nat. Commun..

[31] Shi H. (2020). Highly multiplexed spatial mapping of microbial communities. Nature.

[32] Sheth R.U. (2019). Spatial metagenomic characterization of microbial biogeography in the gut. Nat. Biotechnol..

[33] Richardson M. (2025). SAMPL-seq reveals micron-scale spatial hubs in the human gut microbiome. Nat. Microbiol..

[34] Pietroni C. (2025). Micro-scale spatial metagenomics: revealing high-resolution spatial biogeography of gut microbiomes. bioRxiv.

[35] Bourceau P. (2023). Visualization of metabolites and microbes at high spatial resolution using MALDI mass spectrometry imaging and in situ fluorescence labeling. Nat. Protoc..

[36] Ntekas I. (2026). Spatial transcriptomics maps host–gut microbiome biogeography at high resolution. Nat. Microbiol..

[37] Lötstedt B. (2024). Spatial host–microbiome sequencing reveals niches in the mouse gut. Nat. Biotechnol..

[38] Godron M. (1981). Patches and structural components for a landscape ecology. Bioscience.

[39] DeAngelis D.L., Grimm V. (2014). Individual-based models in ecology after four decades. F1000Prime Rep..

[40] Rahimi S. (2022). A vector-agent approach to (spatiotemporal) movement modelling and reasoning. Sci. Rep..

[41] Wang J. (2025). Individual-based modeling unravels spatial and social interactions in bacterial communities. ISME J..

[42] DeAngelis D.L., Yurek S. (2017). Spatially explicit modeling in ecology: a review. Ecosystems.

[43] Cole E. (2023). Spatial implicit neural representations for global-scale species mapping. arXiv.

